# Salmonella Sternoclavicular Septic Arthritis in a Non-sickle Cell Disease Patient

**DOI:** 10.7759/cureus.34094

**Published:** 2023-01-23

**Authors:** Hind M Alghamdi, Elham A Alghamdi, Sudhir K Chowdhary, Samar AlQahtanii, Reem AlMohaini

**Affiliations:** 1 Orthopedic Surgery, King Saud Medical City, Riyadh, SAU; 2 Surgery, Faculty of Medicine, ‏Imam Mohammad Ibn Saud Islamic University, Riyadh, SAU

**Keywords:** sternoclavicular joint (scj), pathogens, salmonella infection, septic arthritis, sternoclavicular joint (scj) septic arthritis

## Abstract

Septic arthritis is one of the most common orthopedic emergencies. In most cases, the joints affected are large (e.g., knees, hips, and ankles). The presentation of septic arthritis in the sternoclavicular joint (SCJ) has a relatively low prevalence rate, most commonly found in intravenous drug users. *Staphylococcus aureus* is the most common pathogen identified. We report a case of a 57-year-old male with a known case of diabetes mellitus, hypertension, and ischemic heart disease who complained of chest pain and was later found to have right-side SCJ septic arthritis. The procedure involves aspiration of pus using ultrasound guidance as well as irrigation of the right SCJ. The result of a pus culture from the right SCJ (a rare joint to be affected) was Salmonella, which is an atypical infection, specifically in non-sickle cell disease patients. The patient was treated with a specific antibiotic covering this pathogen.

## Introduction

Septic arthritis is a common orthopedic emergency; it is linked to high morbidity, mortality, and great functional impairment if left untreated [1]. The commonly involved joints are large joints (e.g., knees, hips, and ankles), but any joint can be affected [2]. The sternoclavicular joint (SCJ) is a saddle-shaped diarthrodial joint with a central disk. It is the only true articulation between the upper limb and the axial skeleton. The SCJ is stabilized by a strong and complex soft tissue envelope, including its capsule, multiple ligaments, and surrounding muscle groups [3]. Infections of the SCJ account for less than 0.5-1% of all joint infections [4,5]. The presentation of SCJ septic arthritis can present as chest pain radiating down the arm. The low prevalence and ambiguous presentation of SCJ often delay its diagnosis of septic arthritis [6,7]. Currently, no standardized diagnostic and therapeutic algorithms for SCJ infection exist, as defined in the literature [8].

The most common pathogen identified is *Staphylococcus aureus*, which causes half of all SCJ infections, followed by Pseudomonas species. Salmonella infection of the SCJ appears extremely rare, especially in non-sickle cell disease patients [9]. Herein, we report a case of SCJ arthritis in a non-drug user and non-sickle cell disease patient diagnosed with Salmonella infection in the SCJ.

## Case presentation

**Figure 1:**
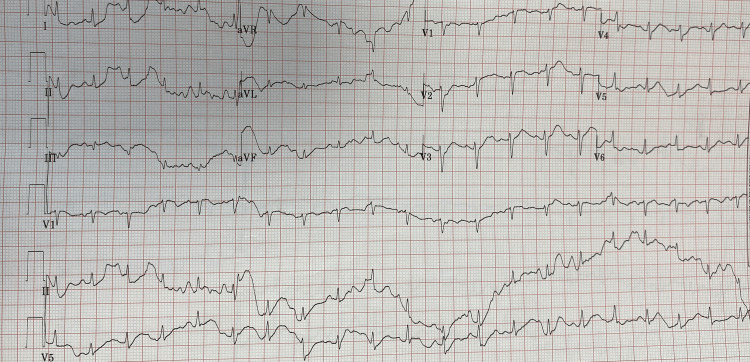
Electrocardiogram (ECG) showing sinus tachycardia with ST-elevation myocardial infarction (STEMI) leading to aVR and VIII.

A 57-year-old male with a known case of diabetes mellitus, hypertension, and ischemic heart disease with a history of percutaneous coronary intervention (PCI) stenting three years before this admission presented to the King Saud Medical City Emergency Department (KSMC-ER) with typical chest pain associated with shortness of breath and sweating for one day. An electrocardiogram (ECG) of the patient revealed sinus tachycardia associated with ST-elevation myocardial infarction (STEMI), which was visible only in leads aVR and VIII (Figure 1).

The impression was a non-ST-elevation myocardial infarction (NSTEMI). The patient was admitted to the cardiology ward for acute chest pain, managed conservatively with antihypertensive medication and aspirin. Once the symptoms improved, the patient became stable.

The patient’s labs revealed hemoglobin (Hb) of 13.3 g/dL, white blood cells (WBCs) of 18.9 x 10^9^/L, and platelets (PLT) of 54 x 10^9^/L. Hematologists were consulted regarding a low platelet count of <30k. Hematology was followed for thrombocytopenia, and they recommended starting an antiplatelet. The patient was prescribed not to go for coronary artery angiography (CAG).

**Figure 2:**
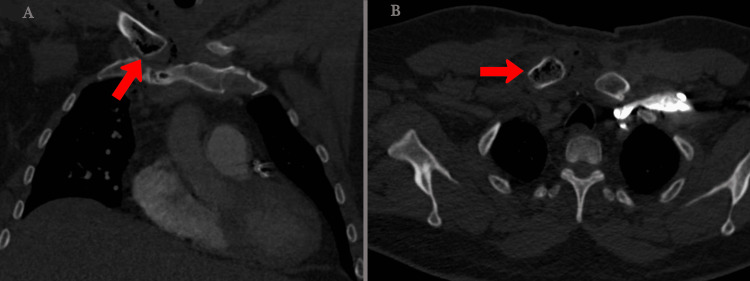
Computed tomography of the chest wall and bones with contrast. The images show (A) coronal cut of computed tomography and (B) axial cut of computed tomography showing right SCJ erosive changes, effusion, soft tissue thickening, and clavicle gas bubbles.

On the third day post-presentation, the patient developed a fever of 39.3°C with chest pain radiating to the neck with local swelling and erythema to the right SCJ. Therefore, further investigation was carried out to rule out other causes of chest pain, such as pulmonary embolism. Computed tomography (CT) of the chest wall and bones with contrast was conducted for the patient and was positive for right SCJ erosive changes, effusion, soft tissue thickening, and clavicle gas bubbles extending into the adjacent anterior mediastinum (Figures 2A, 2B).

Upon further history, the patient stated that the swelling started a few days before admission, localized to the clavicle region, with no other joint involvement. He denied any history of drug abuse or drinking alcohol, recent surgeries, trauma, intraarticular injections, osteoarthritis, sickle cell disease, current respiratory disease, sexually transmitted disease, or any form of infections or history of malignancies.

On examination of the patient, he was on the bed, lethargic, with no open wound, no signs of cellulitis, swelling, and erythema was noted over the right SCJ. There was tenderness and warmth over the right clavicle and supraclavicular area, but there was no obvious bone abnormality. During the upper limb examination, the patient had a painful full, limited range of motion in the right shoulder.

Ultrasound-guided aspiration for the right SCJ was performed by interventional radiology under the aseptic technique. A small amount of pus was aspirated from the right SCJ, then irrigation and aspiration with sterile normal saline. Four samples in four containers were sent for microbiology analysis. There were no immediate post-procedure complications. Synovial analysis showed a classic picture of septic arthritis with Salmonella group D non-typhoidal growth (non-fermenting Gram-negative bacteria).

A septic workup was done with a positive blood culture of Gram-negative bacilli Salmonella group D non-typhoidal, which was sensitive to Augmentin, ciprofloxacin, cefepime, co-trimoxazole, and ceftazidime (third generation cephalosporins). Also, other labs, including results for the hepatitis C virus (HCV), hepatitis B virus (HBV), and human immunodeficiency virus (HIV) labs, were negative.

## Discussion

In contrast to hyaline cartilage, fibrous cartilage lines the SCJ. Some joints, such as the SCJ, pubic symphysis, and sacroiliac joints, are characterized by bulky central disks, which are susceptible to infection [1]. These joints are well known to have high intraarticular pressures due to their restricted distention capacity [2]. The literature has also described some unusual presentations, such as chest pain extending to the shoulder or neck [1]. Most SCJ infections occur unilaterally (95%), predominantly on the right side (60%), as our case presented [1].

Unusual symptoms of this type of pain can be misinterpreted as a heart infarction. In our case, the patient presented with a real myocardial infarction, with non-improving pain, which reached our diagnosis. Other than clinical manifestations, a diagnostic workup is required. An experienced clinician performs ultrasound as a useful diagnostic tool for musculoskeletal conditions, facilitating joint aspiration [10]. Further information can be obtained through a computed tomography (CT) scan with anatomical involvement but cannot show soft tissue involvement, approximately 83% of CT scans are sensitive, which was illustrated in this case and helped in diagnosis [1]. Some risk factors have contributed to some cases, including trauma and surgery, users of intravenous drugs, a history of SLE, use of corticosteroids or other immunosuppressants, neoplasia, and sickle cell anemia, the only risk factor for SCJ infection affected in our patient is a diabetes mellitus (DM) [11]. The study by Branco et al. found that SCJ infection contributed to 18% of all septic arthritis cases among heroin addicts [12], and another study conducted by Lee et al. revealed that 76% of cases were associated with systemic disease [2,13]. Infections of the SCJ account for less than 0.5-1% of all joint infections [4,5], and it is believed that intravenous drug users are most likely to become infected with SCJ [7]. A commonly found isolate in SCJ septic arthritis is *Staphylococcus aureus *which accounts for 44% of all septic arthritis [7]. Atypical agents responsible for septic arthritis include Salmonella, which can mainly be associated with sickle cell anemia and systemic lupus erythematosus [14]. Due to Salmonella's intracellular nature, extracellular antibiotics are ineffective against Salmonella. Cephalosporins of the third generation are usually prescribed [15]. As a rationale in this case, the infectious diseases team was consulted on their antibiotic plan, and they recommended cefotaxime for six weeks, then switched to ceftriaxone. Within six weeks of admission, once the patient started to improve, the discharge plan was to give an outpatient department after four weeks and discharged with oral ciprofloxacin 500 mg twice daily. The patient was seen in the orthopedic clinic, and the infection was resolved with an erythrocyte sedimentation rate (ESR) from 104 to 93 mm/hr and chain reactive protein (CRP) from 12.15 to 3.7 mg/dL, and the patient returned to the full range of motion of the right shoulder. This article describes a rare SCJ infection with the atypical organism (Salmonella).

## Conclusions

It is generally recognized that septic arthritis caused by Salmonella species is a rare disease, and the occurrence in rare and serious joints such as SCJ is mainly seen in intravenous drug users. A major issue in treating general septic arthritis is the identification and isolation of the cause of the disease. Since empirical antibiotics are ineffective against Salmonella, this paper showed how important it is to isolate and correctly identify the etiological agent. Salmonella-related SCI has never been described in other papers in non-sickle cell patients.

## References

[1] Tasnim S, Shirafkan A, Okereke I (2020). Diagnosis and management of sternoclavicular joint infections: a literature review. J Thorac Dis.

[2] Bar-Natan M, Salai M, Sidi Y, Gur H (2002). Sternoclavicular infectious arthritis in previously healthy adults. Semin Arthritis Rheum.

[3] Thompson MA, Barlotta KS (2018). Septic arthritis of the sternoclavicular joint. J Emerg Med.

[4] Guerra C, Spillane LL (1996). Sternoclavicular septic arthritis in a patient with end-stage liver disease. Ann Emerg Med.

[5] Gerscovich EO, Greenspan A (1994). Osteomyelitis of the clavicle: clinical, radiologic, and bacteriologic findings in ten patients. Skeletal Radiol.

[6] Mamarelis G, Sohail MZ, Mamarelis A, Fawi H, Mahaluxmivala J (2017). Spontaneous bilateral sternoclavicular joint septic arthritis and lumbar discitis: an unusual case in a healthy adult. Case Rep Orthop.

[7] Ross JJ, Saltzman CL, Carling P, Shapiro DS (2003). Pneumococcal septic arthritis: review of 190 cases. Clin Infect Dis.

[8] McAninch SA, Smithson C 3rd, Juergens AL, Collins JN, Nanda A (2018). Sternoclavicular joint infection presenting as nonspecific chest pain. J Emerg Med.

[9] Ross JJ, Shamsuddin H (2004). Sternoclavicular septic arthritis: review of 180 cases. Medicine (Baltimore).

[10] Ernberg LA, Potter HG (2003). Radiographic evaluation of the acromioclavicular and sternoclavicular joints. Clin Sports Med.

[11] Morgan MG, Forbes KJ, Gillespie SG (1990). Salmonella septic arthritis: a case report and review. J Infect.

[12] Brancós MA, Peris P, Miró JM (1991). Septic arthritis in heroin addicts. Semin Arthritis Rheum.

[13] Lee JJ, Kim JS, Jeong WS, Kim DY, Hwang SM, Lim SY (2010). A complication of subclavian venous catheterization: extravascular kinking, knotting, and entrapment of the guidewire - a case report. Korean J Anesthesiol.

[14] Uygur E, Reddy K, Ozkan FÜ, Söylemez S, Aydin O, Senol S (2013). Salmonella enteridis septic arthritis: a report of two cases. Case Rep Infect Dis.

[15] Tassinari AM, Romaneli MT, Pereira RM, Tresoldi AT (2019). Septic arthritis caused by Salmonella enterica serotype Rubislaw: a case report. Rev Soc Bras Med Trop.

